# Editorial: Early identification of affective and non-affective psychoses: From psychopathology to biomarkers

**DOI:** 10.3389/fpsyt.2023.1144943

**Published:** 2023-02-06

**Authors:** Laura Fusar-Poli, Andrea Aguglia, Umberto Albert

**Affiliations:** ^1^Department of Brain and Behavioral Sciences, University of Pavia, Pavia, Italy; ^2^Section of Psychiatry, Department of Neuroscience, Rehabilitation, Ophthalmology, Genetics, Maternal and Child Health, University of Genoa, Genoa, Italy; ^3^IRCCS Ospedale Policlinico San Martino, Genoa, Italy; ^4^Department of Medicine, Surgery and Health Sciences, University of Trieste, Trieste, Italy; ^5^Azienda Sanitaria Integrata Giuliano-Isontina - ASUGI, UCO Clinica Psichiatrica, Trieste, Italy

**Keywords:** bipolar disorder, schizophrenia, psychosis, major depressive disorder, prevention, diagnosis

Psychotic disorders—such as schizophrenia or bipolar disorder and related disorders—may severely affect the patients, their families, and the society. The lifetime prevalence of psychotic disorders has been estimated at around 3% in the general population (1) and may raise up to 7% when considering the broad spectrum of psychotic-like experiences (2). Affective and non-affective psychoses may present with heterogeneous clinical features, etiologies, severity, duration, and prognosis. The onset of these conditions can be generally collocated between the second and the third decade of life, even if premorbid changes may be subjectively reported or clinically detected years before the actual onset of psychopathological symptoms (3).

Given the individual, societal, and financial burden, research about the early detection of psychotic disorders has been flourishing over the last decades. Indeed, early identification is essential to implement early intervention, which in turn may improve the clinical outcomes and lead to a better prognosis over the illness course (4). In the Research Topic “*Early identification of affective and non-affective psychoses: From psychopathology to biomarkers*,” we collected a series of papers aimed to increase the knowledge on the early recognition of major psychotic disorders.

The Research Topic included a total of five studies, of which four (Grent-‘t-Jong et al.; Huang et al.; Liu et al.; Sun et al.) were focused on putative biomarkers of psychotic disorders or predictors of disease course. One study (Reis et al.) was instead a case report focused on psychopathological issues.

Huang et al. explored the role of oxidative stress in the etiopathology of schizophrenia. The authors found higher venous pH, PvO_2_, and fasting blood glucose levels and lower superoxide dismutase, lactic acid, and PvCO_2_ levels in patients with schizophrenia compared with healthy controls. Superoxide dismutase was negatively correlated with general psychopathology; furthermore, PvO_2_ levels were closely related to venous pH in schizophrenia and related to PvCO_2_ in the control group. The authors suggested a potential a role of glucose metabolism in the onset of schizophrenia.

Liu et al. examined potential relationship between cognitive functioning and neuroanatomical markers, using Magnetic Resonance Imaging (MRI), in patients with treatment-resistant schizophrenia (TRS) or early-stage schizophrenia and healthy controls. The authors found that individuals with TRS present with significant cognitive impairment and reduction in cortical thickness and subcortical volume compared with individuals in the early stages of the disorder and healthy controls.

Sun et al. aimed to investigate the dynamics of EEG microstates in drug-naïve and first-episode schizophrenia, testing the relationship between EEG microstates and clinical symptoms. Abnormal patterns of EEG microstates were found in drug-naïve, first-episode patients compared with healthy controls. Specifically, results showed that microstate class C had an increased occurrence, duration, and contribution, while microstate class D had a decreased occurrence and contribution. In addition, positive symptomatology, measured using the Positive and Negative Syndrome Scale (PANSS), was negatively correlated with the occurrence of microstate D.

Grent-‘t-Jong et al. used Magnetic Resonance Spectroscopy (MRS) to evaluate gamma-aminobutyric acid (GABA), glutamate, and glutamate-plus-glutamine (Glx) levels in auditory cortex in three groups of participants: people with clinical high-risk for psychosis (CHR-P), people who met criteria for non-psychotic disorders, such as mood and anxiety disorders, eating disorders, and substance use disorder, but not for CHR (CHR-N), and healthy controls. Results did not show robust differences in GABA, glutamate or Glx between clinical groups and controls. However, a specific reduction in Glx was observed in CHR-P compared with CHR-N. Furthermore, across all CHR participants, stronger Glx reductions were linked to greater attenuated psychotic symptoms but did not correlate with symptomatology, cognition, or functioning in CHR-P. These patterns suggested that auditory cortex Glx levels may potentially be useful in differentiating early-stage psychosis from non-psychotic disorders, but not in predicting clinical or functional outcomes.

Finally, Reis et al. illustrated the case of a 17-year-old male who presented to child and adolescent psychiatric services with symptoms of panic disorder, associated with social withdrawal, mild depression, insomnia, and fatigue. Over 6 months, subthreshold psychotic symptoms gradually started, suggesting a prodromal phase of a psychotic disorder. In the case report, the authors discussed the limitations of categorical approach and the potential utility of a transdiagnostic and dimensional approach to better identify patients at risk of developing psychiatric disorders and implement early intervention strategies.

Our Research Topic has confirmed that the study of biomarkers is crucial for the early identification of both affective and non-affective psychoses. To date, research has examined markers across multiple domains, such as genetics (5), electrophysiology (6), neuroimaging (7), neuropsychology (8), and serology (9–11). Nevertheless, while such biomarkers are ideally utilizable in clinical practice, their real-world clinical utility appears still very limited (12). To further enhance the early identification of psychotic disorders, future researchers should focus on the translational applicability and integration of biomarkers in the clinical settings. Candidate markers should be validated in studies with larger samples and conducted using a robust methodology. Also, it is important for putative markers to be easily acquired in the community across multiple settings and, ideally, practical enough to be easily implemented (13).

It is essential to acknowledge that, despite the progress that neuroscientific research has made over recent years, psychopathology still remains a cornerstone for the early identification of psychiatric disorders. In fact, diagnostic processes rely on psychiatric interview skills and clinical reasoning. Thus, a detailed description of clinical and behavioral variables is necessary. In this regard, as discussed in the case report by Reis et al., clinical presentations can be uncertain, thus making the diagnostic process a real challenge for clinicians. As psychotic disorders typically emerge during transitional age, the adoption of a transdiagnostic approach in youths may be helpful in capturing all the situations at-risk and monitoring them over time to improve the outcome (14). Studies integrating the identification of biomarkers with a detailed clinical characterization of patients with affective and non-affective psychoses can promote a substantial advancement in the early diagnosis of psychiatric disorders for personalization of care.

## Author contributions

LF-P wrote the first draft of the manuscript. AA and UA critically revised the manuscript and provided important intellectual contributions. All authors have read and approved the final version.
